# Non-invasive peripheral nerve stimulation selectively enhances speech category learning in adults

**DOI:** 10.1038/s41539-020-0070-0

**Published:** 2020-08-06

**Authors:** Fernando Llanos, Jacie R. McHaney, William L. Schuerman, Han G. Yi, Matthew K. Leonard, Bharath Chandrasekaran

**Affiliations:** 1 Department of Communication Science and Disorders, University of Pittsburgh, Pittsburgh, PA 15260 USA; 2 Neurological Surgery, University of California, San Francisco, San Francisco, CA 94143 USA

**Keywords:** Human behaviour, Peripheral nervous system

## Abstract

Adults struggle to learn non-native speech contrasts even after years of exposure. While laboratory-based training approaches yield learning, the optimal training conditions for maximizing speech learning in adulthood are currently unknown. Vagus nerve stimulation has been shown to prime adult sensory-perceptual systems towards plasticity in animal models. Precise temporal pairing with auditory stimuli can enhance auditory cortical representations with a high degree of specificity. Here, we examined whether sub-perceptual threshold transcutaneous vagus nerve stimulation (tVNS), paired with non-native speech sounds, enhances speech category learning in adults. Twenty-four native English-speakers were trained to identify non-native Mandarin tone categories. Across two groups, tVNS was paired with the tone categories that were easier- or harder-to-learn. A control group received no stimulation but followed an identical thresholding procedure as the intervention groups. We found that tVNS robustly enhanced speech category learning and retention of correct stimulus-response associations, but only when stimulation was paired with the easier-to-learn categories. This effect emerged rapidly, generalized to new exemplars, and was qualitatively different from the normal individual variability observed in hundreds of learners who have performed in the same task without stimulation. Electroencephalography recorded before and after training indicated no evidence of tVNS-induced changes in the sensory representation of auditory stimuli. These results suggest that paired-tVNS induces a temporally precise neuromodulatory signal that selectively enhances the perception and memory consolidation of perceptually salient categories.

## Introduction

Humans are excellent perceptual learners. Yet, a notable and well-documented exception is the acquisition of non-native speech categories in adulthood^1,2^. The significant effort required by adults to learn new speech categories is considered a prime example of how mature sensory and perceptual systems prioritize stability (e.g., processing native speech) over plasticity (e.g., acquiring non-native speech). Recent neuroscience work suggests that it may be possible to overcome limitations in adult plasticity by pairing electrical stimulation of the peripheral nervous system with behaviorally relevant events^3–5^. Here, we examined the impact of transcutaneous vagus nerve stimulation (tVNS), a safe and non-invasive method of peripheral nerve stimulation, on the acquisition of new speech categories in adulthood.

While infants can acquire native speech categories with little or no supervision^6–8^, speech category training studies show that adult learners benefit from some form of supervision^9–14^. In a speech category training task, trial-based corrective feedback induces a reinforcement signal that yields robust and generalizable learning^9,10,13,15,16^. At the neural level, frontal and striatal networks that encode corrective feedback are directly involved in building new speech category representations within the temporal lobes^10,16,17^. In an incidental speech category training task, a task-irrelevant speech signal is synchronized with a task-relevant event (e.g., feedback on videogame performance) to increase learners’ state of arousal during the presentation of the speech signal^11,14,18,19^. Incidental training also results in robust speech category learning and engages the striatal network that modulates the emergence of new speech category representations in speech training tasks driven by corrective feedback^12^. Together, these findings demonstrate that the emergence of new speech category representations in the adult brain is facilitated by reinforcement and arousal systems that modulate perception, memory, and attention.

As we learn more about the systems that modulate the acquisition of new speech categories, it is becoming possible to stimulate these systems non-invasively to improve perceptual behavior in learners. A major advantage for tVNS as a potential neuromodulator of speech category learning is the potential to activate multiple neural systems via afferent connectivity^20–22^. In contrast to neurostimulation approaches designed to modulate localized neural activity^23–25^, vagus nerve stimulation conveys a global diffuse signal to cholinergic and noradrenergic modulators of auditory processing, memory, and attention^3,5,20,22,26–28^. Recent neuroimaging^20,22^ and animal tract-tracing^29^ studies suggest that this global neuromodulatory signal can be initiated non-invasively by applying electrical current to the auricular branch of the vagus nerve, which innervates the outer ear.

**Fig. 1:**
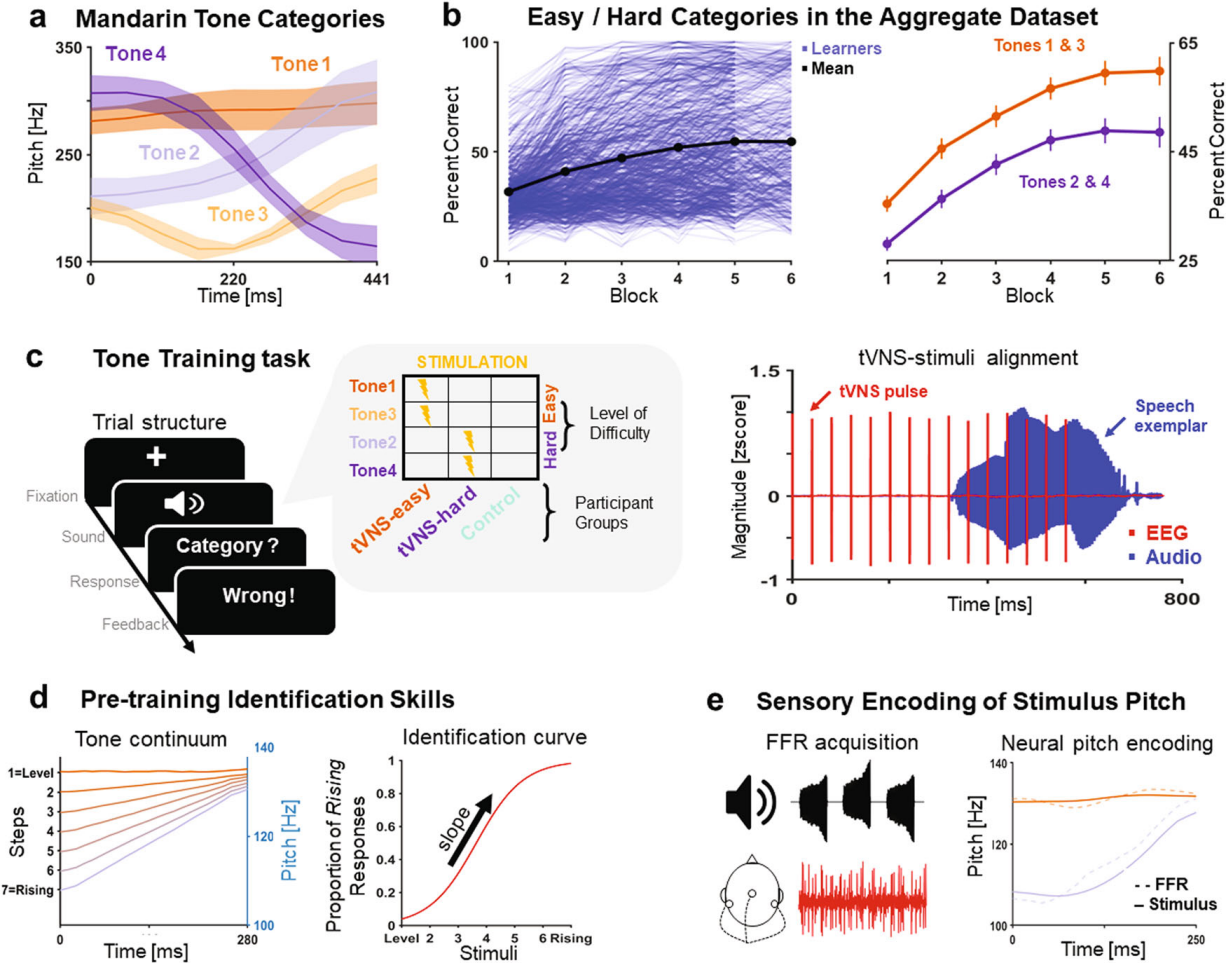
Methods. **a** Pitch contours (M and SD) of the four Mandarin Chinese tones across syllables and female speakers included in the study. **b** To estimate the categories that would be easier (Tone 1 and Tone 3) and harder (Tone 2 and Tone 4) to learn, we examined an Aggregate dataset of 678 Mandarin tone learners collected across eight published training studies. Left. Individual and mean percent correct responses across learners and tone categories. Right. Mean percent correct responses (99% CI) across learners and categories for easier- and harder-to-learn categories. **c** Left. Categorization trial structure and categories paired with stimulation in each participant group. Right. tVNS-stimulus alignment in one example trial. **d** Before the training task, we conducted a perceptual identification task to rule out group differences in perceptual identification skills. Left. Participants were asked to categorize as “rising” or “level” a perceptual continuum of Mandarin tones ranging from high-level (Tone 1) to low-rising (Tone 2) pitch. Right. The slope of the perceptual identification curve was used as a metric of perceptual acuity. **e** Left. To assess the effects of tVNS on the sensory encoding of stimulus pitch, we collected frequency-following responses (FFRs) to Mandarin tones before and after the training task. Right. To assess neural pitch encoding quality, we correlated neural (FFR) and stimulus pitch.

Animal and human studies have shown that pairing sounds with vagus nerve stimulation induces robust, stimulus specific, long-lasting plasticity in the auditory context^5,26,30^. Vagus nerve stimulation can also enhance memory and attention, which are critical for perceptual learning^3,4,31–33^. To assess the impact of tVNS on adult speech category learning, we paired tVNS with non-native speech stimuli in a speech category training task. We trained native English-speaking adults to categorize acoustically different Mandarin Chinese syllables into four Mandarin tone categories as a function of their pitch contour. Mandarin Chinese has four non-neutral syllabic pitch contours (i.e., tones) that change word meaning and are lexically irrelevant in English: high-level (Tone 1), low-rising (Tone 2), low-dipping (Tone 3), and high-falling (Tone 4) tones (Fig. 1a). While Mandarin tones are acoustically distinguishable by relative differences in pitch height (e.g., Tone 1 vs. Tone 3) and pitch direction (e.g., Tone 2 vs. Tone 4), English learners are perceptually more sensitive to relative differences in pitch height. Tone 1 and Tone 3 are acoustically cued by higher and lower pitch values, respectively, and are therefore perceptually more salient for English learners. Thus, for English learners, Tone 1 and Tone 3 are easier-to-learn than Tone 2 and Tone 4 (Fig. 1b).

Based on this distinction between easier- vs. harder-to-learn tone categories, we split participants into two experimental groups that received paired tVNS with either Tone 1 and Tone 3, or with Tone 2 and Tone 4 (Fig. 1c). Stimulation intensity was delivered below the perceptual threshold of each learner. We compared the performance of these two experimental groups with a control group of learners that did not receive stimulation during training. This experimental manipulation allowed us to assess the specificity and extent of generalization in VNS-related behavior and auditory sensory plasticity^34,35^.

Prior speech category training work has demonstrated that changes in arousal induced by performance pressure or selective attention to task-relevant acoustic cues enhances learning^36,37^. Given the expected modulatory effects of electrical stimulation on auditory sensory plasticity and arousal, we predicted that tVNS would enhance speech category learning selectively. Since arousal is argued to selectively enhance attention to stimuli that have greater perceptual salience^27,38,39^, we hypothesized stronger enhancement when tVNS was paired with easier-to-learn categories (Tone 1 and Tone 3). Alternatively, consistent with cue-weighting theories, tVNS paired with difficult-to-learn categories (Tone 2 and Tone 4) may selectively promote greater sensitivity to pitch direction, enhancing the perceptual saliency and learning of this critical feature.

To rule out pre-training differences in perceptual identification skills and auditory sensory encoding between groups, we conducted a perceptual identification task (Fig. 1d) and collected scalp-recorded frequency-following responses (FFRs; Fig. 1e) before the training session. We also collected FFRs after the training session to assess the extent to which tVNS modulated the sensory representation of non-native pitch. Additionally, we measured electrophysiological correlates of VNS in every participant receiving stimulation to assess the extent to which sub-threshold peripheral nerve stimulation evoked brainstem activity supportive of peripheral nerve engagement.

To anticipate, our results demonstrate that speech category learning is enhanced only when tVNS is paired with the speech categories that are easier-to-learn. The learning-related benefits of tVNS emerged rapidly immediately after the first training block (40 trials) and generalized to exemplars from novel talkers. On this short timescale, tVNS did not modulate the sensory representation of Mandarin tones, as measured by the FFR. These results demonstrate that it is possible to enhance adult speech category learning in a highly specific manner by inducing a temporally precise neuromodulatory signal via non-invasive peripheral nerve stimulation.

## Results

We trained 36 native English speakers to categorize natural speech exemplars of the four Mandarin tone categories. Stimuli were presented in six training blocks, and each tone exemplar was presented once per block (see “Speech category training task” in the Methods). On each trial, participants indicated which category they heard and received visual feedback (“Correct” / “Wrong”) following their response (Fig. 1c left). Stimulation intensity was delivered below the perceptual threshold, surrounding the onset of the auditory stimuli (see “Electrical stimulation procedure” in the Methods; Fig. 1c right). Sub-perceptual stimulation thresholds were calibrated on an individual participant basis, using a staircase procedure. The two stimulation groups differed only on whether tVNS was paired with the tone categories that were easier-to-learn (Tone 1 and Tone 3; “tVNS-easy group”) or harder-to-learn (Tone 2 and Tone 4; “tVNS-hard group”). These tone categories were selected on an empirical basis, based on a cohort of 678 English learners of Mandarin tones (Aggregate dataset) collected across eight published studies using no stimulation^9,13,15,16,36,37,40^ (see “Aggregate dataset” in the Methods; Fig. 1b left). The analysis of correct responses by category in the Aggregate dataset revealed that Tone 1 and Tone 3 were easier-to-learn than Tone 2 and Tone 4 (one-way ANOVA: F_2712,3_ = 49.84, *p* < 0.001; post-hoc Tukey adjusted ps < 0.0125; Fig. 1b right). A third participant group (Control group) did not receive stimulation during training but wore the tVNS electrodes and performed the staircase procedure to enable participant blinding. After six training blocks, participants completed a Generalization block in which they categorized new category exemplars produced by novel speakers. In this block, they did not receive tVNS or corrective feedback.

### Effects of tVNS on speech category learning

**Fig. 2:**
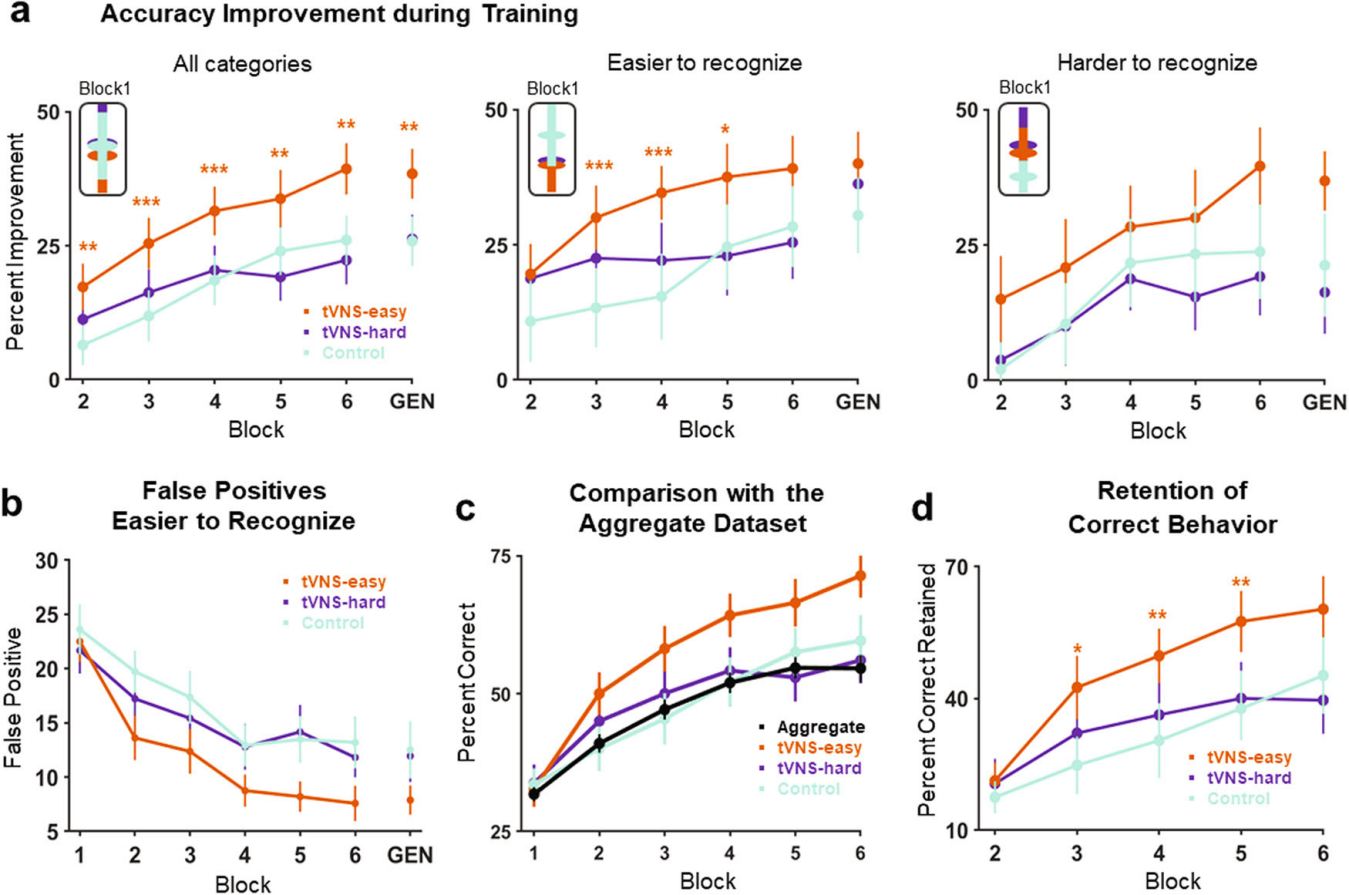
Behavioral results. **a** Left. Percent accuracy improvement (M and SEM) over Block 1 across subjects and categories for each participant group; the Generalization block (Block 7) is denoted as “GEN”. Middle-Right. Percent accuracy improvement (M and SEM) over Block 1 for easier-to-learn (middle) and harder-to-learn (right) categories. The asterisks denote statistical differences for group-by-block interactions (Control group, Block 1 = reference levels) in the following mixed-effects model: response outcome ~ group*block + (1 | subject) + (1 | tone category). **b** Percentage of false positives for Tone 1 and Tone 3 (M and SEM) by group and block. **c** Percentage of correct responses across subjects and categories for each participant group (M and SEM) and the Aggregate learning dataset, consisting of 678 comparable learners receiving no stimulation (M and 99% CI to compensate for the large sample size). **d** Percent of correct trials that were retained from the previous block.

First, we assessed the effects of training in the Control group receiving no stimulation. We conducted a mixed-effects model analysis with a binomial logit link (see “Analysis of categorization accuracy” in the Methods). The dependent variable was the trial-by-trial response outcomes (correct vs. incorrect) of every participant in each group. We found a significant effect of trial for the Control group (*β* = 0.006, *z* = 10.57, *p* < 0.0001; Fig. 2a). This result demonstrates that training was effective in the absence of stimulation.

Next, we tested the central hypothesis that pairing tVNS with specific tone categories would enhance learning. To assess this hypothesis, we examined the group-by-trial interactions in the logit mixed-effects model introduced above (Control group = reference level). We found a positive and significant effect for the tVNS-easy group (*β* = 0.002, *z* = 2.36, *p* = 0.018; Fig. 2a left). This result indicates that the tVNS-easy group exhibited a better trial-by-trial improvement than the Control group. Notably, by the third block the tVNS-easy group had already improved their Block 1 accuracy as much as the Control group did by the last training block (~26% improvement). These results demonstrate that participants learned faster when tVNS was paired with the tone categories that were easier-to-learn (Tone 1 and Tone 3).

In contrast, the group-by-trial interaction for the tVNS-hard group was not significant (*β* = −0.001, *z* = −1.83, *p* = 0.066; Fig. 2a left). This result indicates that the trial-by-trial improvement of the tVNS-hard group was comparable to that of the Control group. Thus, tVNS did not enhance learning when it was paired with the categories that were more difficult to learn (Tone 2 and Tone 4).

Next, we examined whether the effects of stimulation were more accentuated for the subset of tone categories that were paired with tVNS. We fit two logit mixed-effects models (see “Analysis of categorization accuracy” in the Methods). One model was fit with individual trial-by-trial response outcomes for Tone 1 and Tone 3 (easier-to-learn categories), and the other model was fit with the outcomes for Tone 2 and Tone 4 (harder-to-learn categories). The analysis of group-by-trial interactions (Control group = reference level) revealed no significant differences in trial-by-trial improvement between the tVNS-hard and Control groups for any subset of categories (easier-to-learn: *β* = −0.001, *z* = −1.22, *p* = 0.21; harder-to-learn: *β* = −0.001, *z* = −1.40, *p* = 0.15; Fig. 2a right). In contrast, the group-by-trial interaction for the tVNS-easy group was positive and significant only for the subset of categories that were paired with stimulation in this group (easier-to-learn: *β* = 0.002, *z* = 2.13, *p* = 0.032, Fig. 2a middle; harder-to-learn: *β* = 0.001, *z* = 1.12, *p* = 0.26). This result indicates that the learning enhancement found in the tVNS-easy group across training blocks was specific to the set of categories that were paired with stimulation.

Next, we asked whether tVNS improved categorization accuracy in the Generalization block, where participants categorized new speech exemplars without receiving any stimulation or corrective feedback. We fit a logit mixed-effects model (see “Analysis of categorization accuracy” in the Methods) with individual trial-by-block response outcomes in the Generalization block and Block 1. We used Block 1 as reference level to account for *individual* differences in baseline categorization performance. We also used Block 1 to rule out *group* differences in baseline categorization performance at the onset of training. We found a positive and significant group-by-block interaction only for the tVNS-easy group (tVNS-easy: *β* = 0.1, *z* = 3.07, *p* = 0.002; tVNS-hard: *β* = 0.006, *z* = 0.19, *p* = 0.84; Fig. 2a). This result indicates that the learning enhancement found in the tVNS-easy group during the training phase transferred to new category exemplars unpaired with stimulation in the generalization phase. Additionally, group effects were not significant in Block 1 (tVNS-easy: *β* = −0.17, *z* = −0.50, *p* = 0.61; tVNS-hard: *β* = 0.009, *z* = 0.02, *p* = 0.97; Fig. 2a). This result indicates that the learning enhancement exhibited by the tVNS-easy group cannot be attributed to group differences in baseline categorization performance.

During the training phase (Blocks 1–6), the learning enhancement exhibited by the tVNS-easy group was specific to the easier-to-learn categories. However, in the Generalization block, the advantage of the tVNS-easy group over the Control group was slightly larger for harder-to-learn categories. This finding could be due to the confluence of two factors. First, it could be argued that, during the training phase, the Control group also improved their recognition of easier-to-learn categories (relative to harder-to-learn categories). Thus, the initial advantage the tVNS-easy group over the Control group with respect to these categories may have attenuated by the end of the task. Additionally, by the end of the task, the tVNS-easy group may have benefited from a smaller number of false positives for easier-to-learn categories and thus a smaller number of harder-to-learn exemplars miscategorized as easier-to-learn categories. To assess this hypothesis, we examined the number of false positives for easier-to-learn categories in each group. We found that the tVNS-easy group exhibited a larger reduction of these false positives over time (Fig. 2b).

To further demonstrate that there were no group differences in perceptual identification skills relevant for the training task, every participant completed an additional perceptual identification task before the training session (see “Assessment of perceptual identification skills” in the Methods). We included this control because perceptual acuity in this task has been shown to predict individual learning outcomes in our Mandarin tone training paradigm^13^. We found no significant group differences in perceptual acuity (one-way ANOVA: *F*_35,2_ = 0.89, *p* = 0.41). This result indicates that the learning enhancement exhibited by the tVNS-easy group cannot be attributed to group differences in pre-training identification skills that are relevant to succeed in our speech category training task.

### tVNS-related learning enhancement is unlikely to be driven by reward

An interesting possibility is that stimulating easier-to-learn categories is more likely to generate a positive outcome, hence resulting in a reward-based neuromodulatory signal that enhances learning. To test whether tVNS is processed as a reinforcement signal, we created a supplementary experimental condition (tVNS-feedback) wherein tVNS was synchronized with feedback following correct responses in a shorter version of the training task (see Supplementary Materials). To compare the performance of the tVNS-feedback, tVNS-easy, and Control (=reference level) groups, we conducted a mixed-effects model analysis with a binomial logit link. The dependent variable was the trial-by-trial response outcomes (correct vs. incorrect) for every participant. The interaction of trial-by-group for the tVNS-easy group was significant and positive (*β* = 0.006, *z* = 2.8, *p* < 0.005; Supplementary Figure b in Supplementary Materials). In contrast, the interaction of trial-by-group for the tVNS-feedback group was not significant (*β* = 0.001, *z* = 0.59, *p* = 0.55). These results indicate that pairing stimulation with positive feedback (i.e., feedback following correct responses) did not enhance learning. Therefore, the learning enhancement exhibited by the tVNS is unlikely to be driven primarily by a reinforcement-based neuromodulatory signal induced by tVNS.

### Learning enhancement is not predictable from normal learning variation in the Aggregate dataset

Next, we asked whether the learning enhancement exhibited by the tVNS-easy group was within the normal range of variability in the Aggregate dataset (see “Aggregate dataset” in the Methods). We applied non-parametric Monte Carlo sampling statistics to estimate the probability of finding a random sub-population of learners in the Aggregate dataset performing as well as each participant group. Then, we used each of these probabilities as a *p*-value to reject the hypothesis that the performance of the corresponding participant group was representative of the learning variability contained in the Aggregate dataset. While the performances of the tVNS-hard and Control groups were well represented in the Aggregate dataset (*p* = 0.46 and 0.51, respectively), the performance of the tVNS-easy was not (*p* = 0.019; Fig. 2c). This result indicates that the behavioral effects of stimulation on the tVNS-easy group were outside the bounds of normal variation expected from a large and variable population of learners of the same categories.

### Retention of correct stimulus-response associations

Animal and human studies have demonstrated that vagus nerve stimulation can enhance retention and associative memory^4,28,31,32^. Therefore, we assessed whether tVNS enhanced the retention of correct categorization trials between blocks. Specifically, we examined the extent to which tVNS increased the percentage of categorization trials that were correctly categorized on block *n* and on block *n-1* (see “Analysis of retention of correct stimulus-response associations” in the Methods). We fit a linear mixed-effects model with the individual percentages of stimulus trials that were correctly categorized in the current and previous block, starting at Block 2. The group-by-block interaction for the tVNS-hard group was not significant for any block (*p* > 0.05; Fig. 2d). This means that the tVNS-hard and Control groups retained a similar percentage of correct stimulus-response associations between blocks. In contrast, the interaction for the tVNS-easy group was significant for all blocks but the last one (Block 3: *β* = 13.95, *z* = 2.42, *p* = 0.016; Block 4: *β* = 15.52, *z* = 2.69, *p* = 0.007; Block 5: *β* = 16.04, *z* = 2.78, *p* = 0.006; Block 6: *β* = 11.35, *z* = 1.97, *p* = 0.0505 Fig. 2d). This result indicates that, when tVNS was paired with easier-to-learn categories, participants retained a larger proportion of correct categorization responses between most training blocks.

### Sub-perceptual threshold vagus nerve engagement

Most previous work with tVNS has used stimulation intensities just below levels of participant discomfort, but above individual perceptual thresholds. We chose to stimulate below perceptual thresholds to allow participant blinding and this resulted in stimulation intensities that could be several mA lower than what has been used previously in non-invasive work. Therefore, we assessed whether the EEG correlates of sub-threshold tVNS were comparable to those reported for higher stimulation intensities in prior tVNS work (see “Analysis of sub-threshold vagal evoked potentials” in the Methods).

**Fig. 3:**
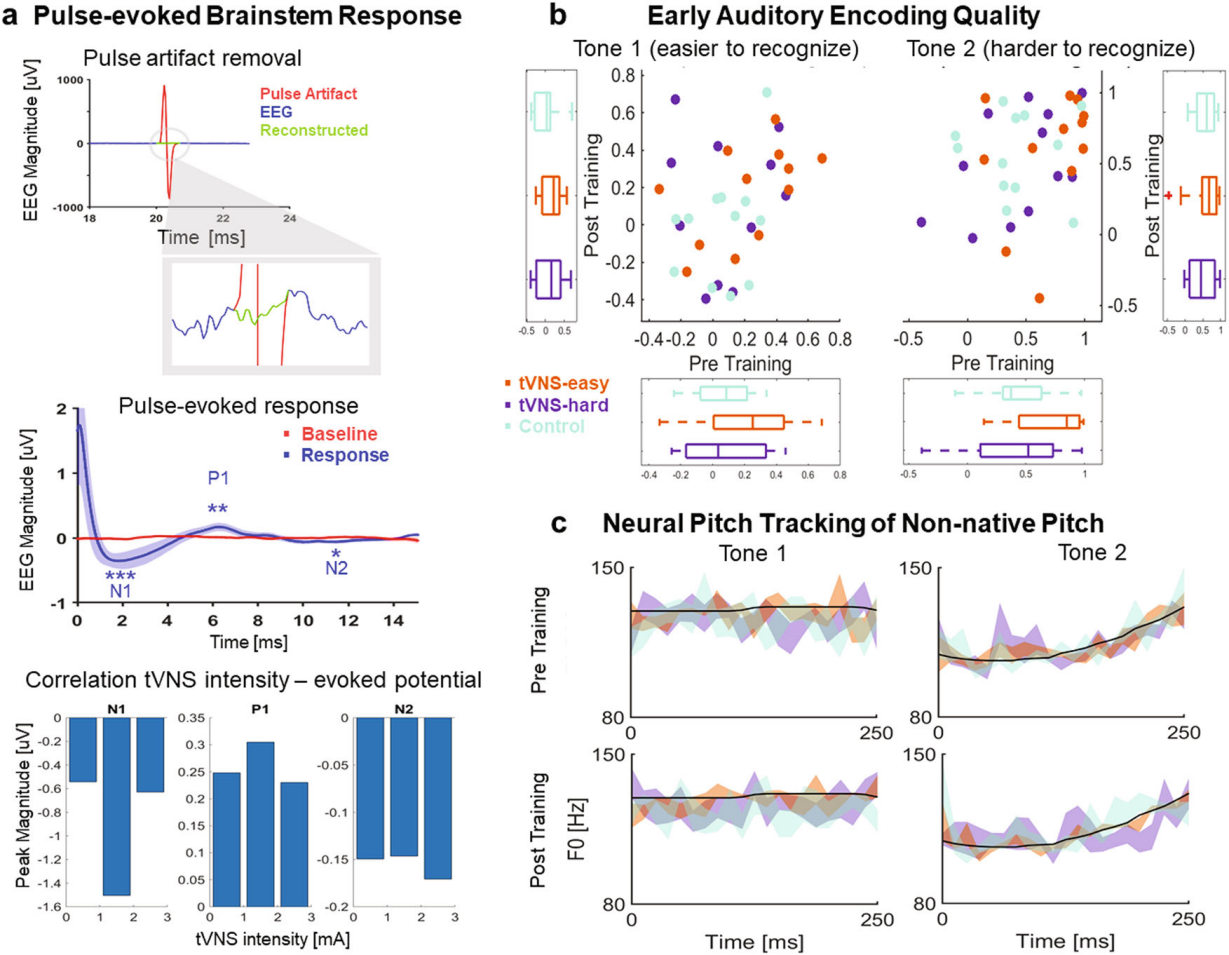
Neural results. **a** Top. Autoregression procedure used to remove tVNS pulse artifacts from the EEG signal. Center. Baseline and sub-threshold vagal evoked potentials (M and SEM) for participants receiving stimulation. The three significant evoked potentials are denoted as N1, P1, and N2. Bottom: inverted-U relationship between tVNS intensity and peak magnitude in the tVNS pulse-evoked response. The bars denote individual pulse-evoked magnitudes averaged for each intensity range (low, intermediate, and high intensities). **b** Stimulus-response correlation coefficients (FFR quality) for each participant, group, and tone before (*x*-axis) and after (*y*-axis) the training session. The panel shows a high degree of individual variability in FFR quality within each group (scatter plots) and no group differences in FFR quality before and after the training session (box plots). **c** Stimulus and neural (FFR) pitch by group (M and SEM) before and after the tVNS session.

Since the vagal evoked potentials reported in the literature^41–43^ arise at brainstem latencies (<15 ms), we collected brainstem electrophysiological responses to tVNS pulses during the training task. After removing stimulation artifacts and averaging the signals within a 15 ms window time-locked to the offset of the tVNS pulse (Fig. 3a top), we found three tVNS pulse-evoked brainstem components with peak magnitudes significantly different from the pre-pulse baseline magnitude: N1 (*M* = −1.07 µV, *t*_40_ = −2.96, *p* = 0.0051), P1 (*M* = 0.27 µV, *t*_40_ = 4.75, *p* < 0.001), and N2 (*M* = −0.15 µV, *t*_34_ = −5.77, *p* < 0.01). The latencies of these peaks, between 2 and 15 ms (Fig. 3a center), were consistent with those reported in prior tVNS work using above-threshold stimulation^41–43^. Together, these results demonstrate that sub-perceptual threshold stimulation causes changes in brainstem electrophysiology that are consistent with peripheral nerve engagement.

To investigate the relationship between the intensity of stimulation and the magnitude of the peak of each component in the pulse-evoked brainstem response, we examined the correlation between individual sensory thresholds and peak magnitudes. We conducted a separate correlation analysis for the peak of each component (i.e., N1, P1, and N2). Pearson’s correlation coefficients were not significant (N1: *r* = 0.051, *p* = 0.82; P1: *r* = −0.16, *p* = 0.49; N2: *r* = 0.047, *p* = 0.83). This result indicates that peripheral nerve engagement, as indexed by tVNS pulse-evoked potentials, did not linearly increase with stimulation intensity. However, we found stronger N1 and P1 peaks for intermediate tVNS intensities (1 mA < intensity ≤ 2 mA), as compared to low (0.2 mA < intensity ≤ 1 mA) and high (2 mA < intensity ≤ 3 mA) intensities. This result suggests that the relationship between tVNS intensity and the pulse-evoked brainstem response follows a non-linear, inverted-U pattern (Fig. 3a bottom).

To examine the extent to which the magnitude of the peaks of the tVNS pulse-evoked brainstem response predicted individual learning improvements, we examined the correlation between peak magnitude and learning improvement across participants. Learning improvement was quantified as the percentage of Block 1 accuracy improved by the last block of training. We conducted a separate correlation analysis for each peak (N1, P1, and N2). We found a trend according to which the greater the improvement, the stronger the peak. However, the trend was not significant (N1: *r* = −0.39, *p* = 0.07; P1: *r* = 0.36, *p* = 0.09; N2: *r* = −0.1, *p* = 0.64).

### Sensory representation of non-native pitch

To examine the effects of brief tVNS exposure on early auditory sensory representations of non-native pitch contours, we collected FFRs to Mandarin Chinese tones before and after the training session (see “Analysis of sensory representation of non-native pitch” in the Methods). The FFR is a scalp-recorded potential that reflects phase-locked activity in cortical and subcortical networks within the auditory system^44–47^. When presented with a sound with harmonic structure, like a Mandarin Chinese tone, the FFR synchronizes in phase to the fundamental frequency (F0) of the sound, providing a reliable neural correlate of pitch kinematics^45^. We collected FFRs to two exemplars of Tone 1 (easier-to-learn) and Tone 2 (harder-to-learn) before (session 1) and after (session 2) the training session. FFR quality was measured with the stimulus-response correlation metric, a well-established metric of sensory pitch encoding in prior FFR and speech training studies^13,15,48^. We conducted a separate linear mixed-effects modeling analysis for each category exemplar. The effects of group and group-by-session interaction were not significant (see Table 1; Fig. 3b). These results indicate that there were no significant group differences in FFR quality before the tVNS session and that the learning enhancement exhibited by the tVNS-easy group was not followed by stimulation-related changes in the sensory representation of non-native pitch.

**Table 1:** No effects of tVNS on sensory encoding quality.

Tone 1 (Easier to learn)						
	tVNS-easy			tVNS-hard		
	*β*	*z*	*p*	*β*	*z*	*p*
Group	0.15	1.38	0.17	0.02	0.19	0.85
Group × session	0.01	0.14	0.88	0.11	0.82	0.41

Tone 2 (Harder to learn)						
	tVNS-easy			tVNS-hard		
	*β*	*z*	*p*	*β*	*z*	*p*
Group	0.27	1.89	0.062	0.01	0.08	0.92
Group × session	−0.26	−1.56	0.12	−0.08	−0.51	0.61

Next, we examined the extent to which the auditory sensory representation of Tone 1 and Tone 2 became more distinct from each other after the tVNS session. Here, we implemented a machine learning classifier^49^ to decode Mandarin tone categories (Tone 1 and Tone 2) from FFRs collected before and after the tVNS session (see “Analysis of sensory representation of non-native pitch” in the Methods). Then, we used the percentage of FFRs that were incorrectly classified to score the degree of confusion between the sensory representations of Tone 1 and Tone 2. We found no significant group differences (one-way ANOVA: *F*_2,34_ = 1.38, *p* = 0.26) in FFR confusion after the tVNS session (tVNS-easy: M = 19.81%, SD = 18.69%; tVNS-hard: 32.03 = 25.04%, SD = 25.04%; Control: M = 31%, SD = 16.62%). To assess group differences with respect to the percentage of confusions that were changed after the tVNS session, we subtracted the confusion scores obtained before the tVNS session from the confusion scores obtained after the tVNS session. We found that the tVNS session had a small impact (<10%) across groups. Specifically, after the tVNS session, mean FFR confusion increased by 9.86% (SD = 13%) in the tVNS-easy group and 7.63% (SD = 17.13%) in the tVNS-hard group. This result shows that in these groups the performance of the classifier got worse after the training session, although by a quite small amount. In the Control group, mean FFR confusion decreased by 1.86 % (SD = 22.98%). This result means that in this group the performance of the classifier improved after the training session, although by a quite small amount. These group differences were not significant (one-way ANOVA: *F*_2,34_ = 1.36, *p* = 0.27). Consistent with the results for FFR quality, this result indicates that the tVNS session did not have a significant impact in the sensory representation of non-native pitch.

## Discussion

We investigated the extent to which pairing non-invasive, sub-perceptual threshold tVNS with behavioral training enhances the ability to categorize non-native speech categories in adults. When tVNS was paired with the speech categories that were easier-to-learn, participants performed significantly better than those who did not receive stimulation. Specifically, participants who received stimulation paired with Tone 1 and Tone 3 learned correct stimulus-response associations faster with accuracy differences emerging immediately after the first block (=40 trials). They also retained a greater proportion of these correct associations between blocks. Crucially, this group-specific learning improvement also generalized to new speech category exemplars presented without accompanying stimulation and corrective feedback. These results demonstrate that tVNS can be used to accelerate speech perceptual learning in humans in a highly specific manner.

We ruled out the possibility that the specificity of our results may be driven by a sampling bias involving a greater distribution of individuals pre-disposed to be successful learners in the tVNS-easy group. We leverage the Aggregate dataset of 678 learners to address this possibility, where individual variability shows that some participants start out with higher accuracy levels than others (Fig. 1b)^9,13,15,16,36,37,40^. At a basic level, the group-specific enhancement observed with tVNS is fundamentally different from normal variability observed across participants who perform the same task without receiving stimulation. We demonstrated this by randomly sampling participant groups from the Aggregate dataset and found that the pattern of performance observed in the tVNS-easy group was not well-represented. This result demonstrates that the tVNS-induced changes in individual learning behavior were independent of endogenous variability. Additionally, the performance of the tVNS-easy group was more accurate than the performance of the Aggregate dataset. In contrast, the performance of the tVNS-hard and Control groups were predictable from the Aggregate dataset. Since the size of the Aggregate dataset was much larger than the size of the participant groups, this finding demonstrates that the differences between the participant groups were not likely due to their sample size.

As indexed by the FFR and the perceptual identification task, the experimental groups did not differ on sensory processing or perceptual identification of tone categories prior to the speech category training procedures. A previous study shows that performance on the identification task is a strong indicator of tone category learning success^13^. Groups also did not differ in performance on the first training block, where participants learn the arbitrary category to button mapping. Instead, group differences emerged after the first block and were not explained by pre-existing group differences.

Together, the results of the present study demonstrate that non-invasive, sub-perceptual threshold VNS can selectively enhance learning of complex, behaviorally relevant speech categories in adult humans. What are the neurophysiological mechanisms that can explain this enhancement effect? We posit that sub-threshold tVNS engages the ascending brainstem network, as indicated by significant vagal evoked potentials that are highly consistent with prior studies examining supra-threshold VNS^41–43^. Thus, some aspect of these neuromodulatory pathways caused a learning enhancement specific to Tone 1 and Tone 3 for the tVNS-easy group. Furthermore, tVNS did not improve the sensory representation of non-native stimulus pitch, as measured with the FFR^44–47^. Prior work has shown that both VNS^5,26^ and longitudinal behavioral training^13^ can independently change the sensory representation of sound properties in the brain. This sensory plasticity has been linked to cholinergic neuromodulation^30,50^. Much of the previous work on perceptual learning has shown that eliciting robust changes to the sensory encoding of fine-grained stimulus properties requires longer training periods than the approximately 25 min utilized in the present study^5,13,34^. Our results indicate that any acceleration in behavioral performance was not associated with rapid changes in sensory plasticity. It is possible that learners in the present study did not have enough time to develop and/or consolidate tVNS-induced changes in sensory plasticity, or that they did not have enough learning experience to improve the representation of unfamiliar fine-grained stimulus properties. This null result, however, indicates that the behavioral changes we observed are not due to fundamental changes to the sensory representation of fine-grained stimulus properties, but instead likely result from processes related to the adjustment of the functional mapping between broad representations of stimulus signals and abstract categories.

While our results indicate that a brief tVNS session is not enough to improve the sensory representation of non-native pitch, it is also possible that our range of intensities for sub-threshold tVNS was not optimal to drive auditory sensory plasticity in auricular and/or non-invasive modalities. Prior invasive VNS work^5,51^ has documented cholinergic-induced plasticity in the primary auditory cortex after stimulating the cervical branch of the vagus nerve with currents between 0.4 mA and 1.6 mA across multiple sessions. We stimulated with intensities varying between 0.2 and 3 mA across participants. The optimal intensity range of intensities for cholinergic activation may differ across VNS modalities, and the precise relationship between cervical VNS and auricular VNS cannot be discerned by the current study.

Prior invasive VNS work has also demonstrated that the effects of VNS in the primary auditory cortex can vary as a function of the combination of stimulation parameters (frequency, intensity, and pulse width). For example, small intensities (e.g., 0.2 mA) are insufficient to drive auditory cortical plasticity when combined with short pulse widths (e.g., 100 μs) and require longer pulse widths (e.g., 500 μs) to drive cortical plasticity in invasive cervical VNS^52^. Using a pulse width of 100 μs, an invasive cervical VNS study^51^ has identified a peak of auditory plasticity at moderate intensity currents around 0.8 mA. Here, the impact of VNS intensity on auditory plasticity followed an inverted-U pattern according to which moderate intensities drove more plasticity than low and high intensities. In our study, we combined a short pulse width of 150 μs with moderate to higher tVNS intensities varying across participants between 0.2 mA and 3 mA. Notably, we found relatively stronger peaks in the pulse-evoked brainstem response when we stimulated with unextreme intensity currents between 1 mA and 2 mA.

Prior invasive VNS work^53^ has also demonstrated that the frequency of stimulation can influence neural activation in the locus coeruleus. While the total number of driven spikes in response to a VNS train is similar at most stimulation frequencies, higher stimulation frequencies can result in greater maximal discharge rates over a shorter duration. Prior invasive VNS work has also reported a slight but significant reduction in neural spikes at 120 Hz compared to moderate frequencies around 30 Hz. While we do not think it is currently possible to draw direct links between invasive cervical VNS in animal models and auricular tVNS in humans, we used this prior literature to run pilot experiments to ensure that similar combinations of stimulation parameters could be used safely and effectively in our paradigm. This led us to use a stimulation frequency of 25 Hz. Since we did not manipulate the frequency of stimulation in the present study, we cannot assess the effects of this parameter in our VNS modality (i.e., transcutaneous auricular VNS). This remains an open area for future research.

A route to VNS-induced changes in learning and memory is via noradrenergic modulation. The activation of locus coeruleus (LC) via the afferent vagal system means that VNS can increase arousal and attention. Arousal-based accounts, like the arousal-biased competition (ABC) model^38^ and the Glutamate Amplifies Noradrenergic Effects (GANE) model^52^ argue that temporally precise changes to arousal can enhance the gain of perceptually salient stimuli, resulting in greater memory consolidation specifically for these stimuli. Such arousal-related effects are not found for perceptually less salient stimuli. In the present work, pitch height is the dimension that distinguishes the easier-to-learn Tones 1 and 3 and is a dominant perceptual dimension for native English listeners^53,54^. It is therefore possible that tVNS increases arousal and, when synchronized to more perceptually salient categories, changes the robustness of the emerging representation. Indeed, prior neuroimaging work^10,16^ indicates that category representations emerge in the temporal lobe within a few hundred trials of sound-to-category training, and that the robustness of category representations is category-specific. This account is also consistent with the social gating hypothesis^55^ of speech learning that places significant emphasis on attention and arousal in native language acquisition.

Another interesting possibility is that stimulating easy categories is more likely to generate a positive outcome, hence resulting in a reward-based neuromodulatory signal that enhances learning. To test whether tVNS is processed as a reinforcement signal, we ran an additional condition wherein tVNS was delivered only during correct responses to potentially enhance correct stimulus-response pairing in a shorter version of the sound-to-category training. We did not find significant learning-related enhancement related to tVNS presented during the feedback phase. We therefore posit that the enhancement in the tVNS-easy condition is unlikely to be driven by an interaction between VNS and reward-related neuromodulatory signals induced by positive outcomes. However, given that the tVNS-feedback group received on average 30% less stimulation than the other groups, it is also possible that this group needed more stimulation trials in order to change their learning performance.

Our results also provide a novel perspective on the debate regarding the extent to which explicit vs. incidental feedback is optimal for speech learning in adulthood^11,13,19,34^. Explicit feedback is shown to enhance speech learning^13,15^. Incidental training approaches (for example, video game-based training) can robustly increase speech category learning success^14,18^. Mechanistically, this effect is linked to an endogenous reinforcement signal that activates the striatum^12^. However, video games also modulate arousal and attention, which may also be a gateway to enhanced category learning success.

Together, our results demonstrate that non-invasive transcutaneous vagus nerve stimulation in humans can enhance speech category learning in a highly specific manner. These findings provide further evidence that peripheral neuromodulation may be a useful tool for augmenting behavioral and perceptual paradigms, including higher-level cognitive tasks such as speech sound learning. Together with rigorously tested training paradigms, tVNS may allow adults, who lack the neural plasticity characteristic of early childhood, to achieve substantially better outcomes in challenging tasks like learning a new language.

## Methods

### Ethics

Participants were monetarily compensated for the duration of the experiment and provided written informed consent to take part in the study. The study was approved by the Institutional Review Board of the University of Texas at Austin.

### Participants

We recruited 36 adult native speakers of English (20 females; age: M = 21.60, SD = 3.56) who were unfamiliar with Mandarin Chinese. Since professional music experience can enhance learning performance in our training task^40^, we excluded professional musicians. None of the participants reported any history of hearing problems or neurodevelopmental disorders, and their audiograms revealed normal pure-tone detection thresholds (from 250 to 8000 Hz, octave steps), less than 25 dB for air conduction in each ear. At the beginning of the experiment, participants were randomly assigned to one of three participant groups: tVNS-easy (*N* = 12), tVNS-hard (*N* = 12), and Control (*N* = 12). Prior work has shown that music training influences speech processing^56–58^. Therefore, all the participants completed a music training experience questionnaire before the experiment. The number of years of music experience did not differ significantly across participant groups: tVNS-easy (M = 2 years, SD = 2.49 years), tVNS-hard (M = 1.63 years, SD = 2.15 years), and Control (M = 2.50 years, SD = 5.52 years) (one-way ANOVA: *F*_2,34_ = 0.17, *p* = 0.84). Furthermore, the number of years of music experience observed in the participant groups was significantly smaller than the amount of music experience previously shown to be required to enhance learning in our training task^40,58^ (>10 years).

### Speech category training task

Stimuli consisted of five Mandarin Chinese syllables (/bu/, /di/, /lu/, /ma/, and /mi/), pronounced by four native speakers of Mandarin Chinese (two females). The speakers pronounced each syllable four times, each with a different Mandarin Chinese tone, resulting in a total of 80 speech stimuli (5 syllables × 4 talkers × 4 tones). During the training part, half of the stimuli (*N* = 40; two talkers) were presented in six blocks where each stimulus was played once per block. Participants indicated the tone category on each trial via button press on a keyboard (none of the buttons visually indicated pitch). Immediately following the button press, they were given feedback via visual (“Correct” / “Wrong”) text on a computer screen for 1 s. Immediately following the sixth training block, participants completed a Generalization block. In this block, they categorized the other half of the stimuli (*N* = 40), consisting of the same syllables pronounced by two new talkers. Participants did not receive feedback or stimulation in this block. To avoid physical interference with the stimulation electrodes placed on the left ear (see “Electrical stimulation procedure” in the Methods), the audio was delivered monaurally through the right ear with an insert earphone (ER-3; Etymotic Research, Elk Grove Village, IL).

We confirmed that Tone 1 and Tone 3 were easier to identify than Tone 2 and Tone 4 with a two-sample *t*-test input with the individual percentages of correct categorization responses for each subset of categories (Tone 1 and Tone 3: M = 58.92% correct, SEM = 3.1; Tone 2 and Tone 4: M = 46.42% correct, SEM = 3.84; two-sample *t*-test: *t*_70_ = 2.52, *p* = 0.013).

### Electrical stimulation procedure

To stimulate the vagus nerve non-invasively, we targeted the cymba concha and cymba cavum of the outer ear, which have been shown to be innervated by the auricular branch of the vagus nerve^49^. We delivered current transcutaneously to these sites at amplitudes below each participant’s perceptual threshold. Sub-threshold stimulation avoids evoking somatosensory responses that alert participants to the timing of stimulation. Furthermore, animal models suggest that low-to-mid amplitude stimulation levels are more effective modulators of neural plasticity^51^.

The participant’s left ear was first cleaned with alcohol and abrasive gel using a cotton swab. Silicon putty was then molded to the shape of the participant’s ear. Two Ag-AgCl disc electrodes (4 mm diameter) were embedded in the putty at areas corresponding to the cymba concha (cathode) and cymba cavum (anode) and covered with a salt-free conductive gel. The mold was reinserted into the ear and pressed into place. Electrical stimulation was generated with a BIOPAC STMISOLA Constant Current Isolated Linear Stimulator. Stimulation waveforms consisted of 15 biphasic square-wave pulses (150 μs pulse width) delivered at a rate of 25 Hz^59^ with an amplitude no higher than 3 mA due to safety restrictions. The biphasic waveforms were generated using Matlab (Mathworks, v. 2017a) and transmitted to the stimulator via a National Instruments USB-6211 DAQ card.

Before the speech training session, we used a 0.1 mA-up/0.3 mA-down staircase procedure to identify the perceptual threshold in every participant. The threshold was calculated as the average stimulation amplitude after eight reversals^60^. In the speech training session, stimulation was delivered with a pulse amplitude of 0.2 mA below the participant’s perceptual threshold. A two-sample *t*-test revealed no significant differences in pulse amplitude (*t*_22_ = 1.26; *p* = 0.21) between the two participant groups targeted with stimulation (tVNS-hard: M = 1.67 mA, SD = 0.79 mA; tVNS-easy: M = 1.24 mA, SD = 0.88 mA). The pulse train began approximately 300 ms prior to the onset of the auditory stimulus and continued for 250 ms through approximately half of the auditory stimulus. This tVNS-stimulus alignment spans a variety of alignments reported in prior VNS work^6,27^.

### Analysis of categorization accuracy

To examine the effects of tVNS on speech category learning, we conducted a mixed-effects analysis with binomial logit link^61^. The dependent variable was the trial-by-trial response outcomes (correct vs. incorrect) of each participant in all training blocks^9,37^. The model incorporated fixed effects of group (tVNS-easy, tVNS-hard, and Control = reference level), trial (1 to 240), group-by-trial interactions, and random intercepts of subject and tone category: outcome ~ group*trial + *(1* | *subject) + (1* | *tone category)*. This model provided optimal deviance compared to alternative versions of the model that included random slopes for subject (group | subject) and/or tone category (group | category).

To examine the effects of tVNS on the specific subsets of categories that were paired and unpaired with stimulation in each group, we conducted two mixed-effects analyses with binomial logit link. The dependent variables were the trial-by-trial response outcomes to Tones 1 and 3 in one model, and to Tones 2 and 4 in the other model. The models incorporated the mixed and random effects introduced above.

To assess group differences in categorization accuracy in the Generalization block, we conducted a mixed-effects analysis with a binomial link function. The dependent variable was the trial-by-block response outcomes (correct vs. incorrect) of each participant in the Generalization block and Block 1. The model incorporated fixed effects of group (tVNS-easy, tVNS-hard, and Control = reference level), block (Generalization block, Block 1 = reference level), group-by-block interactions, and random intercepts of subject and tone category: outcome ~ group*block + *(1* | *subject) + (1* | *tone category)*. We used Block 1 to account for *individual* differences in baseline categorization performance, and to test *group* differences in baseline categorization performance at the onset of training.

### Assessment of perceptual identification skills

Before the training session, participants were asked to identify as “level” or “rising” a series of pitch contours ranging between Tone 1 and Tone 2 (Fig. 1d). The slope of the perceptual identification boundary provided by this task (Fig. 1d right) predicted individual learning outcomes in a published Mandarin tone training study using our training paradigm^13^. In this study, speech learners with more categorical, or steeper, perceptual boundary slopes learned faster than learners with less categorical slopes.

Stimuli were created from one Mandarin Chinese syllable acoustically manipulated to span seven pitch steps between a high-level (Tone 1) and low-rising (Tone 2) Mandarin tone^62^ (Fig. 1d left). Tone offsets were fixed to the same pitch value (130 Hz) and tone onsets ranged from 102.08 Hz (T2) to 130.00 Hz (T1) in steps of 3.88 Hz. This step size was adopted to elicit strong categorical identification curves from minimal acoustic differences in native speakers of Mandarin Chinese^13^. Participants were instructed to categorize, without receiving feedback or stimulation, each step of the continuum as “level” or “rising” in 20 randomized blocks where each step was repeated once per block. Sounds were binaurally delivered using the equipment reported in the section “Speech category training task” in the Methods.

The slope of the perceptual boundary between “rising” and “level” categories was computed as the absolute value of the beta coefficient of a logistic-regression curve fit with the proportion of “rising” responses across pitch steps (Fig. 1d right). We assessed group differences in perceptual identification slope with a one-way ANOVA with group and slope as independent and dependent variables, respectively.

### Aggregate dataset

The Aggregate dataset was collected from eight Mandarin tone training studies published since 2014^9,13,15,16,36,37,40,63^. The studies differed minimally in feedback type, feedback delay, performance pressure, and selective attention to pitch patterns. We aggregated categorization responses from a total 678 English-speaking adults matching our participant inclusion criteria. As with our participant groups, they were presented with trial-by-trial corrective feedback and highly variable stimuli. None of the subjects in the Aggregate dataset received stimulation during training. Because most of these subjects lacked a Generalization block, we recovered five to six training blocks—depending on the study—of 40 trials per block. Subjects with a percentage of correct responses higher than 85% in Block 1 were excluded. Following this exclusion, Block 1 accuracy in the Aggregate dataset (M = 31.87% correct) was comparable to that observed in the current study (M = 33.3% correct).

To estimate the chance of finding an Aggregate sub-population with a training performance comparable to that of each of our participant groups, we calculated the mean accuracy improvement across blocks for each subject in the Aggregate dataset and in our participant groups. Next, we created a non-parametric distribution of mean accuracy improvements by sampling one thousand sub-populations of twelve randomly selected subjects from the Aggregate dataset and computing the mean of each random sub-population. The random sub-population size was chosen to match the size of our participant groups. Finally, we calculated the proportion of the non-parametric distribution that was above the mean accuracy improvement of each of our participant groups. We used each proportion as *p*-value to reject the hypothesis that the performance of the corresponding participant group was inside from the margins of normal variation in the Aggregate dataset.

### Analysis of retention of correct stimulus-response associations

To investigate the effects of tVNS on the retention of correct stimulus-response associations across blocks, we calculated the percentage of training trials (*N* = 40; 4 categories × 2 talkers × 5 syllables) that were correctly categorized on block *n* and on block *n-1*. We started with Block 2 and excluded the Generalization block because it contained different stimuli than the training blocks. Then, we fit a linear mixed-effects model with individual retention percentages by block as the dependent variable. The model incorporated fixed effects of group (tVNS-easy, tVNS-hard, and Control = reference level), block (2‒6; block 2 = reference level), interaction of group-by-block, and random intercepts of subject: *retention* ~ group*block + (1|*subject*).

### Analysis of sub-threshold vagal evoked potentials

To assess sub-threshold vagal evoked potentials, we recorded EEGs during the training session from all participants receiving stimulation (BrainVision actiCHAMP system; 25 kHz). EEGs were collected with three Ag-AgCl scalp electrodes (impedance < 5 kΩ) connected to a BrainVision preamplifier (50 dB gain) from the vertex (active), left mastoid (ground) and right mastoid (reference). They were off-line band-pass filtered with a zero-phase second-order Butterworth filter roughly reflecting the phase-locking limitations of neurons in the brainstem^44^ (80 Hz–1 kHz). Each tVNS pulse left a characteristic square-wave artifact in the EEG (Fig. 3a top). We used these artifacts to estimate the onset and offset of each tVNS pulse by cross-correlating a template of the pulse artifact with the EEG. Predicted and observed pulse markers were visually inspected for validation. To avoid ringing artifacts caused by the interaction of pulse artifacts with the band-pass filter, we removed all pulse artifacts before filtering the signal and used the Matlab function *fillgaps.m* to reconstruct the gaps from nearby values (2 ms both sides the gap; Fig. 3a top). The baseline and vagal evoked responses included in our analyses were extracted from EEG segments preceding (baseline responses) or following (vagal evoked responses) the reconstructed gaps.

Vagal evoked responses (0–15 ms after pulse offset) were baseline corrected to the mean voltage of their baseline response (−15–0 ms before pulse onset) and corrected responses with magnitudes exceeding the range of ±35 μV were rejected. Clean responses were averaged for each participant receiving stimulation with the exception of three participants providing unreliable stimulation markers in the EEG signal. Participant responses elicited three clear evoked potentials peaking at approximately 2 ms (N1), 6 ms (P1), and 11 ms (N2) after the pulse offset (Fig. 3a center). We conducted three two-sample t-tests to test whether the magnitude of each evoked potential across participants was significantly different from their corresponding magnitudes in the baseline response.

### Analysis of sensory representation of non-native pitch

FFRs were recorded, digitized, and collected with the equipment, software, and electrode montage used to collect vagal evoked potentials (see “Analysis of sub-threshold vagal evoked potentials” in the Methods). We followed a standard FFR acquisition procedure^13,45^. Single-trial FFRs were elicited with 1100 repetitions of each exemplar, binaurally delivered with an inter-stimulus interval randomly jittered between 122 and 148 ms. Participants were instructed to ignore the audio and focus on a silent movie of choice. EEG was band-pass filtered from 80 Hz to 1 kHz with a zero-phase second-order Butterworth filter. Then, single-trial FFRs were segmented from the EEG channel using a neural latency of 7 ms following the stimulus onset and a temporal window spanning the duration of the evoking stimulus. Single-trial FFRs were baseline corrected to the mean voltage of the noise floor (−40 ms–0 ms) and corrected responses with amplitudes exceeding the range of ±50 μV were rejected. Unrejected trials were averaged for each participant, category (Tone 1/Tone 2), and session (pre-training / post-training) leading to a total of 144 averaged responses (36 participants × 2 categories × 2 sessions). One of these averaged responses was excluded from the analysis because its averaging size (*N* = 686) was extraordinarily below average (M = 995 trials; SD = 37).

To measure pitch encoding quality, we computed the Pearson correlation coefficient (*r*) between the pitch contours of the averaged response and the evoking stimulus. This metric (stimulus-to-response correlation) has been used to evaluate the robustness of subcortical encoding of pitch kinematics as a function of long-term auditory experience^45,48^ and auditory training^13,15,64,65^. Pitch contours were estimated with the autocorrelation method, using a sliding window of 40 ms with 30 ms sliding overlap^13,15,49^. The two linear mixed-effect models incorporated fixed effects of group (tVNS-easy, tVNS-hard, Control = reference level), session (post-training, pre-training = reference level), group-by-session interactions, and random intercepts of subject: r ~ group*session + *(1* | *subject)*.

We also implemented a machine learning classifier, based on the hidden Markov model (HMM), to decode Mandarin tone categories (i.e., Tone 1 vs. Tone 2) from the FFRs collected before and after the tVNS session. Consistent with a prior study we ran a separate HMM for each participant^49^. Training, testing, and cross-validating parameters (training size = 500; testing size = 500; FFR averaging size = 200) were informed by the results of a prior FFR study using the HMM to decode Mandarin tones from FFRs^49^.

### Reporting summary

Further information on experimental design is available in the Nature Research Reporting Summary linked to this paper.

## Supplementary information

SUPPLEMENTARY MATERIALS

Reporting summary check list

## Data Availability

The datasets generated during and/or analyzed during the current study are available from the corresponding author on reasonable request.

## Code availability

The scripts generated during and/or analyzed during the current study are available online: https://zenodo.org/record/3871880#.XtU2mTpKg2w (10.5281/zenodo.3871880).
