# Supplementary material for: Non-invasive peripheral nerve stimulation selectively enhances speech category learning in adults
Source: NPJ Sci Learn. 2020 Aug 6;5:12. doi: 10.1038/s41539-020-0070-0 (PMC7410845; doi:10.1038/s41539-020-0070-0)
Supplement: Supplementary file 1 — SUPPLEMENTARY MATERIALS [file 41539_2020_70_MOESM1_ESM.pdf]

SUPPLEMENTARY MATERIALS

Feedback-locked tVNS. To further examine the mechanisms underlying the impact of tVNS on speech category learning, we collected data from an additional group of participants (N = 10). Here we asked whether tVNS synchronized with feedback on positive outcomes can enhance learning and stimulus-to-response associations. Participants in the tVNS-feedback condition were recruited using similar criteria as the original experiment and underwent identical tVNS thresholding procedures as the other groups. Participants performed a shorter version (120 trials) of the sound-to-category training procedure. The main difference compared to the main experimental conditions (tVNS-easy, tVNS-hard) was that sub-threshold tVNS was delivered during feedback presentation instead of stimulus presentation (**Supplementary Figure a**). Other than the synchronization of the pulses to the corrective feedback (only during ‘correct’ responses’), the stimulation parameters were identical to tVNS-easy and tVNS-hard conditions.

We fit a logit mixed effects model to categorization responses by trial (1 to 120) to compare the learning performance of the tVNS-feedback group relative to the tVNS-easy and control groups in the original experiment. The dependent variable was the trial-by-trial response outcomes (correct vs. incorrect). The model incorporated mixed effects of group (Control = reference level), trial (1 to 120), group-by-trial interactions, and random intercepts of subject and tone category: *outcome ~ group*trial + (1 | subject) + (1 | tone category)*. While pairing tVNS with easy-to-learn categories did enhance trial-by-trial performance relative to Control (p = 0.005), pairing tVNS with positive feedback did not (p = 0.5). For visualization purposes, the **Supplementary Figure b** shows trial-by-trial accuracy within a running window of 40 trials centered on each trial (1 to 120). The number of trials paired with stimulation in the tVNS-feedback group was M = 41.80 (SD = 14.06). The number of trials paired with stimulation in the

tVNS-easy and tVNS-hard group was 60. Thus, the tVNS-feedback group received, on average, 30 % less stimulation than the tVNS-easy.

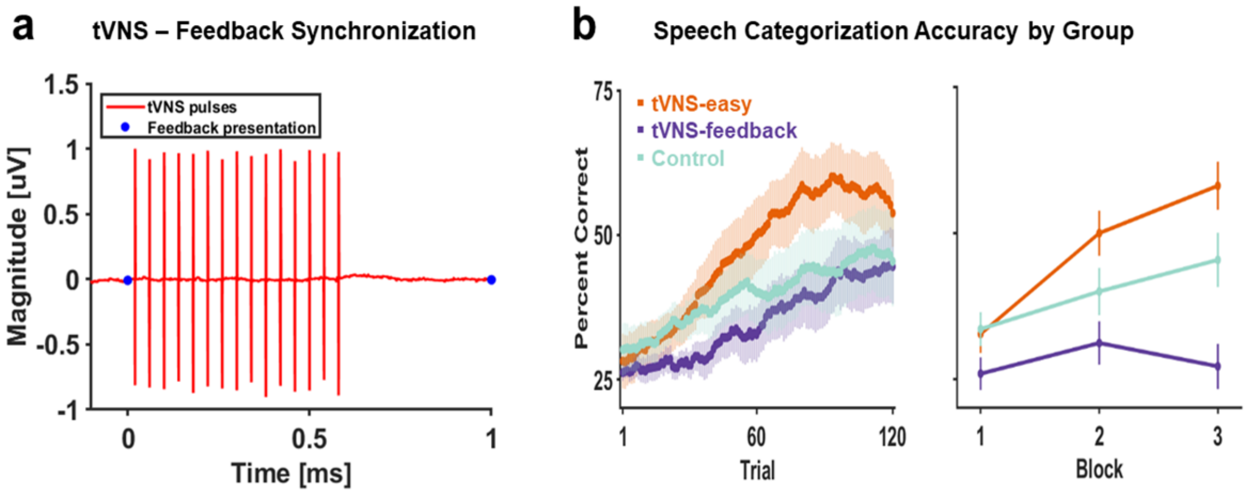

Supplementary Figure. tVNS feedback condition. (a) Synchronization of tVNS and feedback presentation. The solid line shows the tVNS pulse train in one example trial, as reflected by its artifact in the EEG. The two dots mark the onset and offset times for feedback presentation. Feedback onset time was synchronized with the participant’s response after a correct response. **(b)** Trial-by-trial accuracy (left) and by-block accuracy (right) for each group (M and SEM). Trial-by-trial accuracy was obtained with a running window of 40 trials centered on each trial (from 1 – 120 trials).
